# Preoperative Prediction Power of Imaging Methods for Microvascular Invasion in Hepatocellular Carcinoma: A Systemic Review and Meta-Analysis

**DOI:** 10.3389/fonc.2020.00887

**Published:** 2020-06-26

**Authors:** Jiacheng Huang, Wuwei Tian, Lele Zhang, Qiang Huang, Shengzhang Lin, Yong Ding, Wenjie Liang, Shusen Zheng

**Affiliations:** ^1^Department of Hepatobiliary and Pancreatic Surgery, The First Affiliated Hospital, College of Medicine, Zhejiang University, Hangzhou, China; ^2^Collaborative Innovation Center for Diagnosis and Treatment of Infectious Diseases, The First Affiliated Hospital, College of Medicine, Zhejiang University, Hangzhou, China; ^3^Key Lab of Combined Multi-Organ Transplantation, Ministry of Public Health, The First Affiliated Hospital, College of Medicine, Zhejiang University, Hangzhou, China; ^4^College of Information Science & Electronic Engineering, Zhejiang University, Hangzhou, China; ^5^Department of Radiology, The First Affiliated Hospital, College of Medicine, Zhejiang University, Hangzhou, China

**Keywords:** hepatocellular carcinoma, microvascular invasion, radiomics, conventional image, functional image, meta-analysis

## Abstract

**Background:** To compare the predictive power between radiomics and non-radiomics (conventional imaging and functional imaging methods) for preoperative evaluation of microvascular invasion (MVI) in hepatocellular carcinoma (HCC).

**Methods:** Comprehensive publications were screened in PubMed, Embase, and Cochrane Library. Studies focusing on the discrimination values of imaging methods, including radiomics and non-radiomics methods, for MVI evaluation were included in our meta-analysis.

**Results:** Thirty-three imaging studies with 5,462 cases, focusing on preoperative evaluation of MVI status in HCC, were included. The sensitivity and specificity of MVI prediction in HCC were 0.78 [95% confidence interval (CI): 0.75–0.80; *I*^2^ = 70.7%] and 0.78 (95% CI: 0.76–0.81; *I*^2^ = 0.0%) for radiomics, respectively, and were 0.73 (95% CI: 0.71–0.75; *I*^2^ = 83.7%) and 0.82 (95% CI: 0.80–0.83; *I*^2^ = 86.5%) for non-radiomics, respectively. The areas under the receiver operation curves for radiomics and non-radiomics to predict MVI status in HCC were 0.8550 and 0.8601, respectively, showing no significant difference.

**Conclusion:** The imaging method is feasible to predict the MVI state of HCC. Radiomics method based on medical image data is a promising application in clinical practice and can provide quantifiable image features. With the help of these features, highly consistent prediction performance will be achieved in anticipation.

## Introduction

Hepatocellular carcinoma (HCC), causing 781,631 deaths in 2018 worldwide, ranks the fourth cause of death among various cancers (1). Meanwhile, HCC accounts for 4.7% of new cancer cases and is one of the common neoplastic diseases that endanger human health (1). It is well-known that hepatectomy and liver transplantation are the curative therapies for patients with non-advanced HCC (2). Unfortunately, about half of the HCC patients suffer from postoperative recurrence in a few years, resulting in surgical failure and poor prognosis (3). Microvascular invasion (MVI) is a histopathologic evidence of local aggression and is considered as a strong predictor of postoperative recurrence in HCC (3–6). Based on the above research evidences, the *2017 Guidelines for Diagnosis and Treatment of Liver Cancer in China* have required the HCC pathological diagnosis to supply the description of MVI status based on pathological examination and necessary immunohistochemical staining (7).

Preoperative evaluation of the MVI status of HCC will apparently contribute to the development of treatment strategy and prognosis stratification of patients at the same clinical stage, but is still a challenge. Given the clinical significance of MVI status, clinical investigators have attempted to assess the status of preoperative MVI of HCC. Uncommonly, needle biopsy including fine needle aspiration cytology and needle core biopsy were used for preoperative evaluation of liver mass in some specific cases (8). Yet, preoperative biopsy is an invasive examination that may cause concomitant complication or tumor seeding (9). Therefore, it remains disputed whether biopsy can be used to evaluate the MVI status of HCC. Hence, there is an unmet clinical need to preoperatively evaluate the MVI status of HCC.

Medical imaging evaluation plays an irreplaceable role in preoperative evaluation of HCC and can provide clinicians with valuable information (e.g., position, size, and clinical stage of tumors) for decision making. Various research based on preoperative images of HCC was performed to evaluate the MVI status of HCC, but showed no consensus. At first, researchers attempted to correlate the morphological features of tumor images with the MVI status of HCC by comparing the image features with the pathological MVI results of HCC (10–12). Also, quantitative parameters of functional imaging studies were used to evaluate the MVI status of HCC (13–15). Recently, radiomics, defined as the tumor radiomics features extracted through logistic regression as well as machine learning algorithms, has been proposed, which is effective to predict tumor phenotype (16). In a few studies, preoperative images of HCC were used to generate radiomics signatures and construct models to predict the MVI status of HCC (17–19). These different image-based evaluation methods include non-quantitative or objective parameters, univariate or multivariate, small- or large-scale cohorts, and the performance varies in the MVI status evaluation of HCC.

Timely analysis of different image-based evaluation methods is necessary to meet the urgent need for individualized diagnosis and treatment of differential MVI status in clinical practice. In this study, we are aiming to perform a diagnostic meta-analysis to compare the preoperative prediction capability for MVI status of HCC between radiomics and non-radiomics (conventional image, functional image) methods.

## Materials and Methods

### Literature Retrieving

PubMed, Embase, and Cochrane Library were comprehensively searched by applying the following keywords: [(microvascular invasion) OR (MVI)] AND [(malignan^*^ OR cancer OR tumor OR tumor OR neoplas^*^ OR carcinoma) AND (hepatocellular OR liver OR hepatic OR HCC)]. The deadline of our retrieval was Nov. 20, 2019. After elimination of duplicate articles, the abstracts of all remaining literature were reviewed. When it was ambiguous to decide the inclusion of an article only by abstract, full publication was downloaded and reviewed. All studies were screened independently by three authors (JH, TW, and LZ). Discussion was conducted if disagreement about inclusion occurred.

### Selection Criterion

Inclusion criteria were as follows: (1) diagnosis of HCC by pathologic criteria; (2) determination of MVI by pathologic diagnosis; (3) numbers of MVI-positive and MVI-negative being three at least; (4) conduct of computed tomography (CT), magnetic resonance imaging (MRI), or ultrasonic examination before hepatectomy, or liver transplantation; (5) imaging analysis based on radiomics and non-radiomics; and (6) clinical data or pathological reports being inaccessible to reviewers of image analyzing.

Exclusion criteria were as follows: (1) preoperative reception of systemic chemotherapy, transarterial chemoembolization, percutaneous ethanol injection, and radiofrequency ablation; (2) number of MVI-positive or MVI-negative being zero; and (3) evaluation of only clinical characteristics for MVI status prediction in HCC patients without imaging features.

### Data Extraction and Quality Assessment

Numbers of true positive (TP), true negative (TN), false positive (FP), and false negative (FN) were computed according to the numbers of MVI-present, MVI-absent, sensitivity, and specificity reported in the individual studies included. The reference formulas were as follows: sensitivity = TP/(TP+FN), and specificity = TN/(FP+TN). If there were at least two models based on the same group of patients in one study, the model with higher diagnostic accuracy was included into our meta-analysis. QUADAS-2 scale (20) in Revman 5.3 (Cochrane Library Software, Oxford, UK) was measured to appraise the quality of the studies included.

### Statistical Analysis

The pooled sensitivity, specificity, positive likelihood ratio (PLR), and negative likelihood ratio (NLR) were computed in Meta-DiSc 1.4 (Clinical Biostatistics Unit, Ramony Cajal Hospital, Madrid, Spain) (21). Forest plots were visualized on the ggplot2 package in R 3.6.1. Cochrane's *Q*-test and *I*^2^ were considered to detect the heterogeneity among the included studies, and *I*^2^ > 50% indicated the presence of heterogeneity. Meanwhile, summary receiver operating characteristic (sROC) curve was drawn, and then the area under the curve (AUC) was computed to exhibit the diagnostic value of the combined studies (22). The AUC of 0.5–0.7, 0.7–0.9, and >0.9 indicate low, moderate, and high diagnostic power, respectively.

## Results

### Literature Selection and Quality Assessment

**Figure 1:**
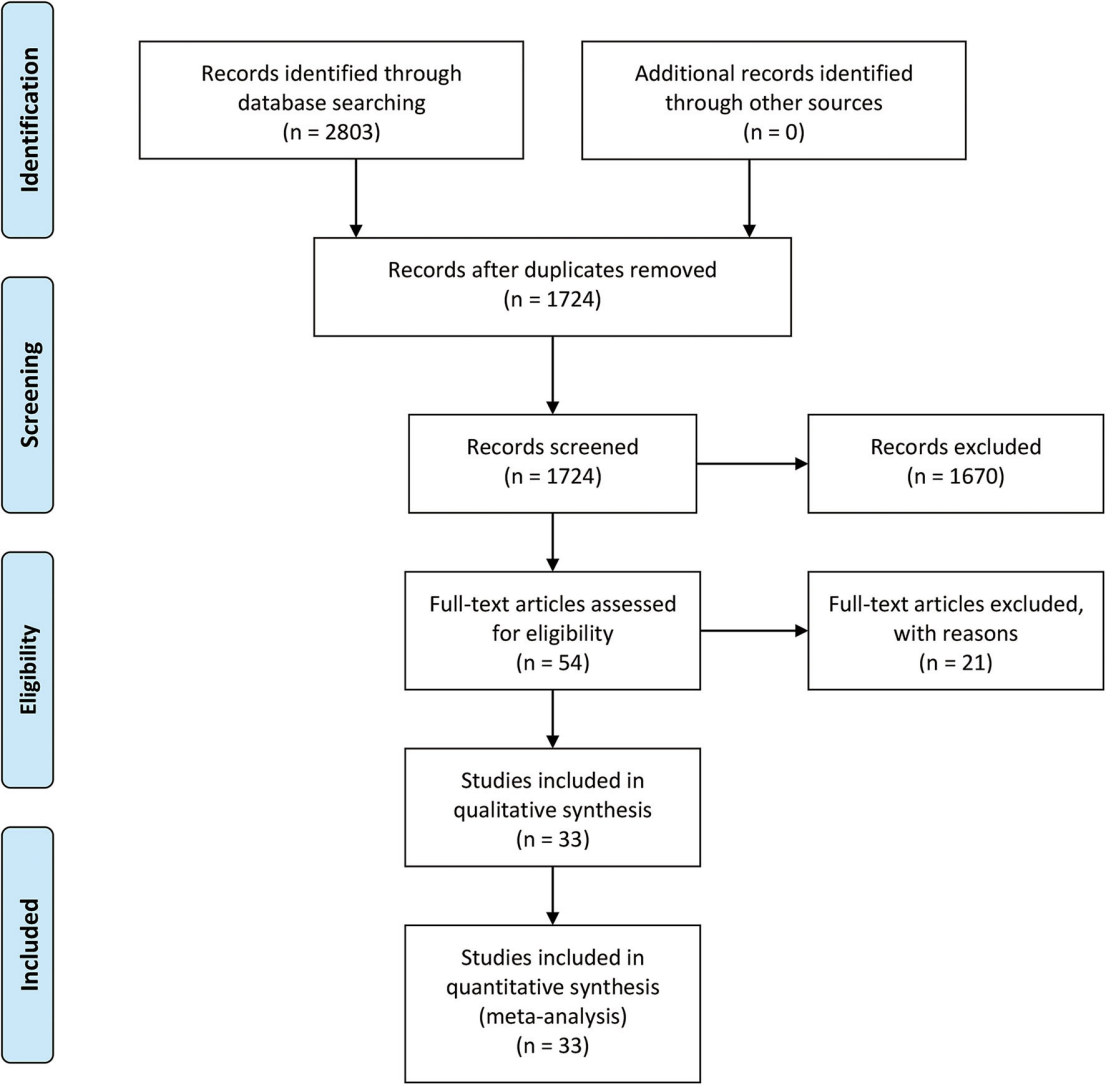
The PRISMA flowchart of the selection procedure.

**Figure 2:**
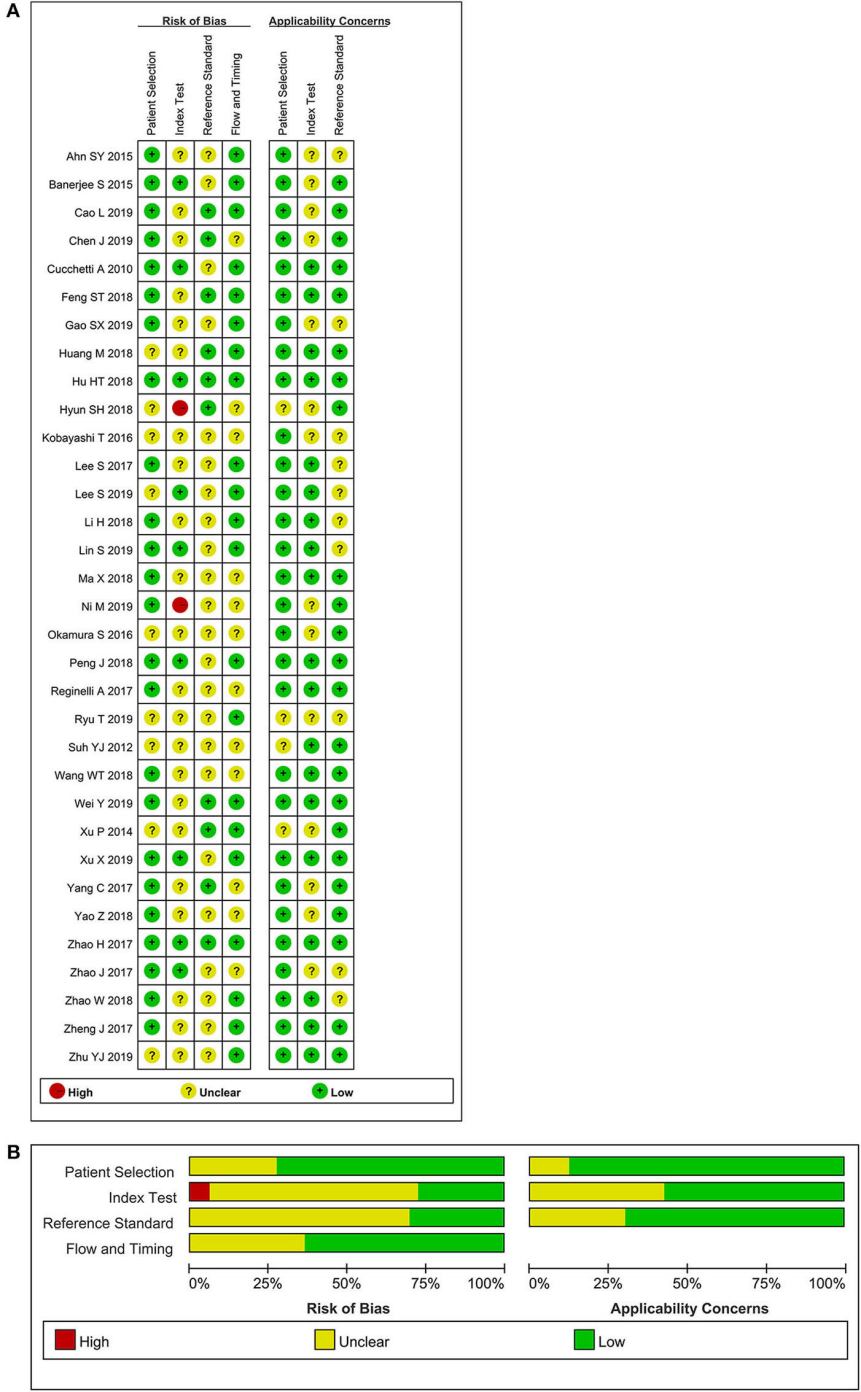
Methodological quality assessment of the included studies based on the QUADAS-2 scale. **(A)** Individual studies, **(B)** summary.

The selection procedure is shown in Figure 1 (23). In total, 33 studies involving nine studies based on radiomics (17–19, 24–29) and 24 studies based on non-radiomics (10–15, 30–47) were eligible for this diagnostic meta-analysis. A total of 5,462 HCC patients were included. Among them, 2,284 patients were pathologically diagnosed as MVI-positive and 3,178 as MVI-negative. The basic characteristics of the 33 included studies are shown in Tables 1, 2, and more details are displayed in Table 3. Figure 2 displays the quality assessment of the included studies based on QUADAS-2 scale.

**Table 1:** Baseline of the included studies based on radiomics in this diagnostic meta-analysis.

**References**	**MVI+HCC**	**MVI–HCC**	**TP**	**FP**	**FN**	**TN**	**Cohort detail**
Zheng et al. (17)	19	32	15	9	4	23	Tumor size ≤ 5 cm
Zheng et al. (17)	34	35	20	4	14	31	Tumor size>5 cm
Peng et al. (24)	127	57	101	16	26	41	Training cohort
Peng et al. (24)	74	46	56	9	18	37	Validation cohort
Xu et al. (19)	100	250	88	58	12	192	Training/validation cohort
Xu et al. (19)	49	96	44	20	5	76	Test cohort
Ma et al. (18)	37	73	28	9	9	64	Training dataset
Ma et al. (18)	18	29	9	7	9	22	Validation dataset
Zhu et al. (29)	37	62	30	13	7	49	Training cohort
Zhu et al. (29)	16	27	13	4	3	23	Validation cohort
Ni et al. (28)	23	35	19	5	4	30	
Feng et al. (26)	42	68	60	10	8	32	Training cohort
Feng et al. (26)	20	30	27	5	3	15	Validation cohort
Hu et al. (27)	136	205	88	47	48	158	Training cohort
Hu et al. (27)	59	82	40	23	19	59	Validation cohort
Yao et al. (25)	21	22	19	3	2	19	

*TP, True positive; TN, True negative; FP, False positive; FN, False negative*.

**Table 2:** Basic characteristic of the included studies based on non-radiomics in this diagnostic meta-analysis.

**References**	**Subgroup**	**MVI+HCC**	**MVI–HCC**	**TP**	**FP**	**FN**	**TN**	**Cohort detail**
Banerjee et al. (10)	Conventional image	45	112	34	7	11	105	
Lee et al. (11)	Conventional image	63	134	33	10	30	124	
Gao et al. (45)	Conventional image	28	32	22	4	6	28	
Cao et al. (43)	Functional image	38	36	26	9	12	27	
Chen et al. (44)	Conventional image	64	85	39	23	25	62	
Lee et al. (12)	Conventional image	78	198	54	5	24	193	Derivation cohort
Lee et al. (12)	Conventional image	23	78	15	11	8	67	External validation cohort
Wei et al. (15)	Functional image	55	80	43	20	12	60	
Lin et al. (46)	Conventional image	106	151	88	47	18	104	Training cohort
Lin et al. (46)	Conventional image	34	66	28	29	6	37	Validation cohort
Ryu et al. (47)	Conventional image	51	60	31	4	20	56	
Li et al. (41)	Functional image	21	20	17	3	4	17	
Zhao et al. (42)	Functional image	18	33	17	12	1	21	
Huang et al. (40)	Conventional image	17	43	9	3	8	40	
Hyun et al. (13)	Functional image	76	82	61	33	15	49	
Wang et al. (14)	Functional image	40	52	28	12	12	40	
Zhao et al. (39)	Functional image	211	107	132	37	79	70	
Reginelli et al. (36)	Conventional image	32	69	28	8	4	61	
Zhao et al. (38)	Conventional image	77	129	62	19	15	110	Derivation cohort
Zhao et al. (28)	Conventional image	39	64	32	11	7	53	Validation cohort
Yang et al. (37)	Functional image	44	92	8	4	36	88	
Okamura et al. (35)	Functional image	33	40	25	9	8	31	
Kobayashi et al. (34)	Functional image	9	51	8	9	1	42	
Ahn et al. (33)	Functional image	14	45	11	9	3	36	
Xu et al. (32)	Functional image	39	70	26	15	13	55	
Suh et al. (31)	Functional image	31	36	29	10	2	26	
Cucchetti et al. (30)	Conventional image	127	48	113	8	14	40	Training cohort
Cucchetti et al. (30)	Conventional image	59	16	55	3	4	13	Testing cohort

*TP, True positive; TN, True negative; FP, False positive; FN, False negative*.

**Table 3:** Clinical characteristics of the included 33 studies.

**Author**	**Modeling methods**	**Imaging modality**	**Feature number**	**Feature type**
Zheng et al. (17)	Logistic regression	CT	162	38 ACM and 128 LBP features
Peng et al. (24)	LASSO regression and logistic regression	CT	18	13 clinical and 5 CT image
Xu et al. (19)	Adjusted OR regression and logistic regression	CT	31	19 clinical and 12 CT image
Ma et al. (18)	SVM, LASSO regression and logistic regression	CT	655	Eight clinical and 647 radiomics
Zhu et al. (29)	Logistic regression	MRI	66	Seven clinical and 59 texture features
Ni et al. (28)	LASSO and GBDT	CT	1,044	Texture features
Feng et al. (26)	LASSO regression and logistic regression	MRI	1,044	Image features
Hu et al. (27)	LASSO regression and logistic regression	Ultrasound	1,044	Radiomics features
Yao et al. (25)	Iterative SR method and SVM	Ultrasound	1,024	MR image
Banerjee et al. (10)	Cox's proportional hazard models	CT	13	Clinical features
Lee et al. (11)	Logistic regression	MRI	23	14 clinical and 9 MR image
Gao et al. (45)	Logistic regression	CT	29	Clinical features
Cao et al. (43)	Logistic regression	MRI	15	12 clinical and 3 DKI
Chen et al. (44)	Cox's proportional hazard models and logistic regression	MRI	19	Clinical features
Lee et al. (12)	Logistic regression	MRI	20	13 clinical and 7 MR image
Wei et al. (15)	Logistic regression	MRI	25	17 clinical and 8 MR image
Lin et al. (46)	Logistic regression	MRI	21	8 clinical and 13 MR image
Ryu et al. (47)	Logistic regression	MRI	21	Clinical features
Li et al. (41)	Logistic regression	MRI	8	Clinical features
Zhao et al. (42)	Logistic regression	MRI	8	Clinical features
Huang et al. (40)	Logistic regression	MRI	8	Radiomics features
Hyun et al. (13)	Logistic regression	PET-CT	12	Clinical features
Wang et al. (14)	Logistic regression	MRI	20	12 clinical and 8 radiomics
Zhao et al. (39)	Logistic regression	MRI	NA	
Reginelli A	Logistic regression	CT	10	Clinical features
Zhao et al. (38)	Logistic regression	CT	NA	Clinical and radiomics features
Yang et al. (37)	Logistic regression	MRI	16	6 clinical and 10 radiomics
Okamura et al. (35)	Logistic regression	MRI	12	Clinical features
Kobayashi et al. (34)	Logistic regression	PET-CT	16	Clinical features
Ahn et al. (33)	Logistic regression	PET-CT	18	3 clinical and 15 PET-CT image
Xu et al. (32)	Logistic regression	MRI	16	8 clinical and 8 MR image
Suh et al. (31)	Logistic regression	MRI	8	Clinical features
Cucchetti et al. (30)	ANN and logistic regression	CT or MRI	15	Clinical features

*LASSO, Least Absolute Shrinkage and Selection Operator; OR, Odds ratio; SVM, Support Vector Machine; GBDT, Gradient boosting decision tree; SR, Sparse representation; ANN, Artificial Neural Network; ACM, Angle co-occurrence matrices; LBP, Local binary patterns; DKI, Diffusion kurtosis imaging*.

### Radiomics for Preoperative MVI Evaluation in HCC

**Figure 3:**
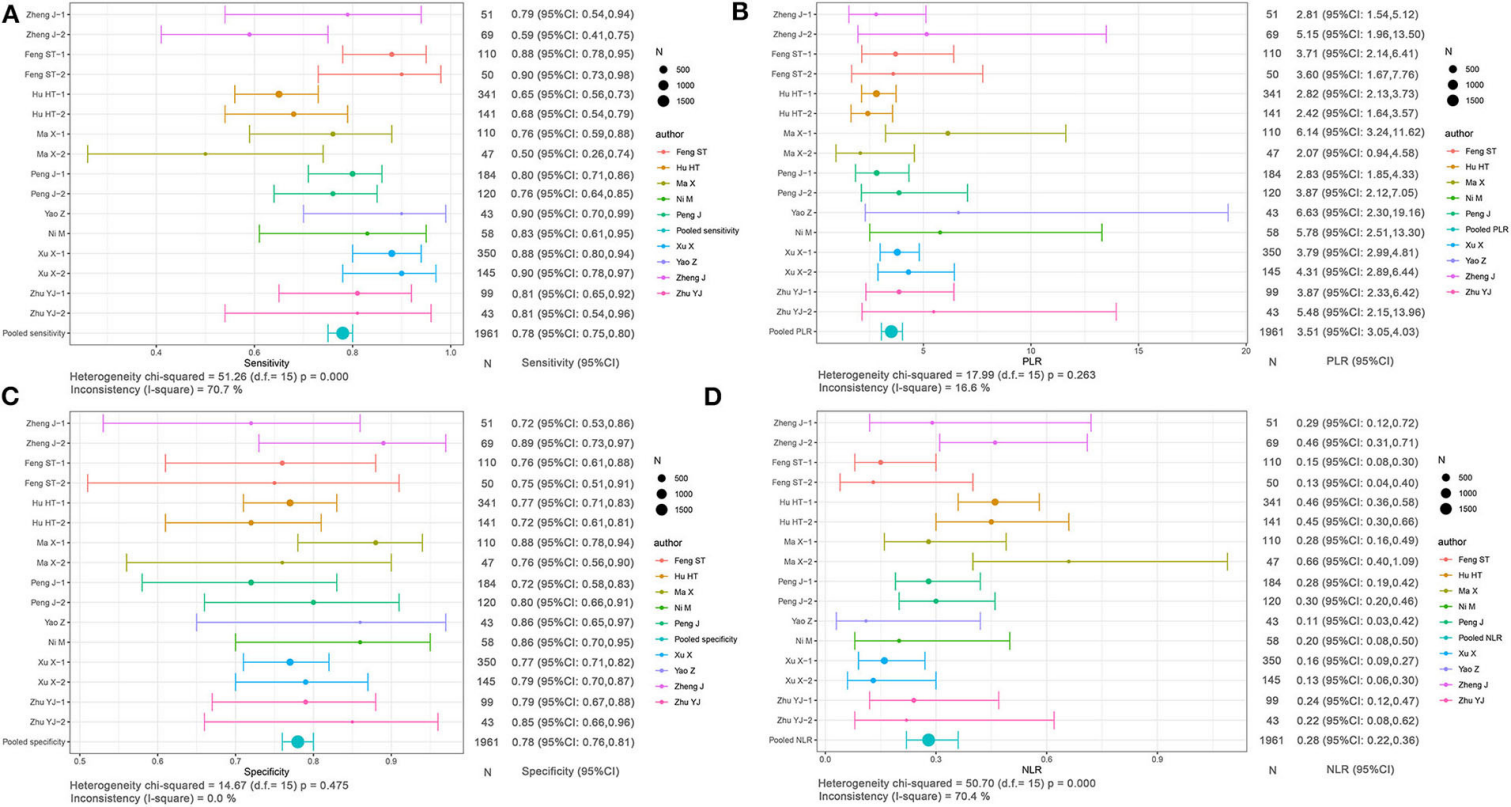
Forest plots based on radiomics for preoperative prediction of MVI in HCC. **(A)** Sensitivity, **(B)** PLR, **(C)** specificity, and **(D)** NLR.

Based on radiomics, there were 1,961 HCC patients, including 812 MVI-present and 1,149 MVI-absent. The diagnostic meta-analysis forest plots and combined results are manifested in Figure 3, Supplementary Table 1. The integrated sensitivity, specificity, PLR, and NLR of the radiomics group were 0.78 [95% confidence interval (CI): 0.75–0.80, *I*^2^ = 70.7%; Figure 3A], 0.78 (95% CI: 0.76–0.81, *I*^2^ = 0.0%; Figure 3C), 3.51 (95% CI: 3.05–4.03, *I*^2^ = 16.6%; Figure 3B), and 0.28 (95% CI: 0.22–0.36, *I*^2^ = 70.4%; Figure 3D), respectively. The AUC based on the sROC curve was 0.8550 (**Figure 7A**), which showed moderate diagnostic value.

### Non-radiomics for Preoperative MVI Evaluation in HCC

**Figure 4:**
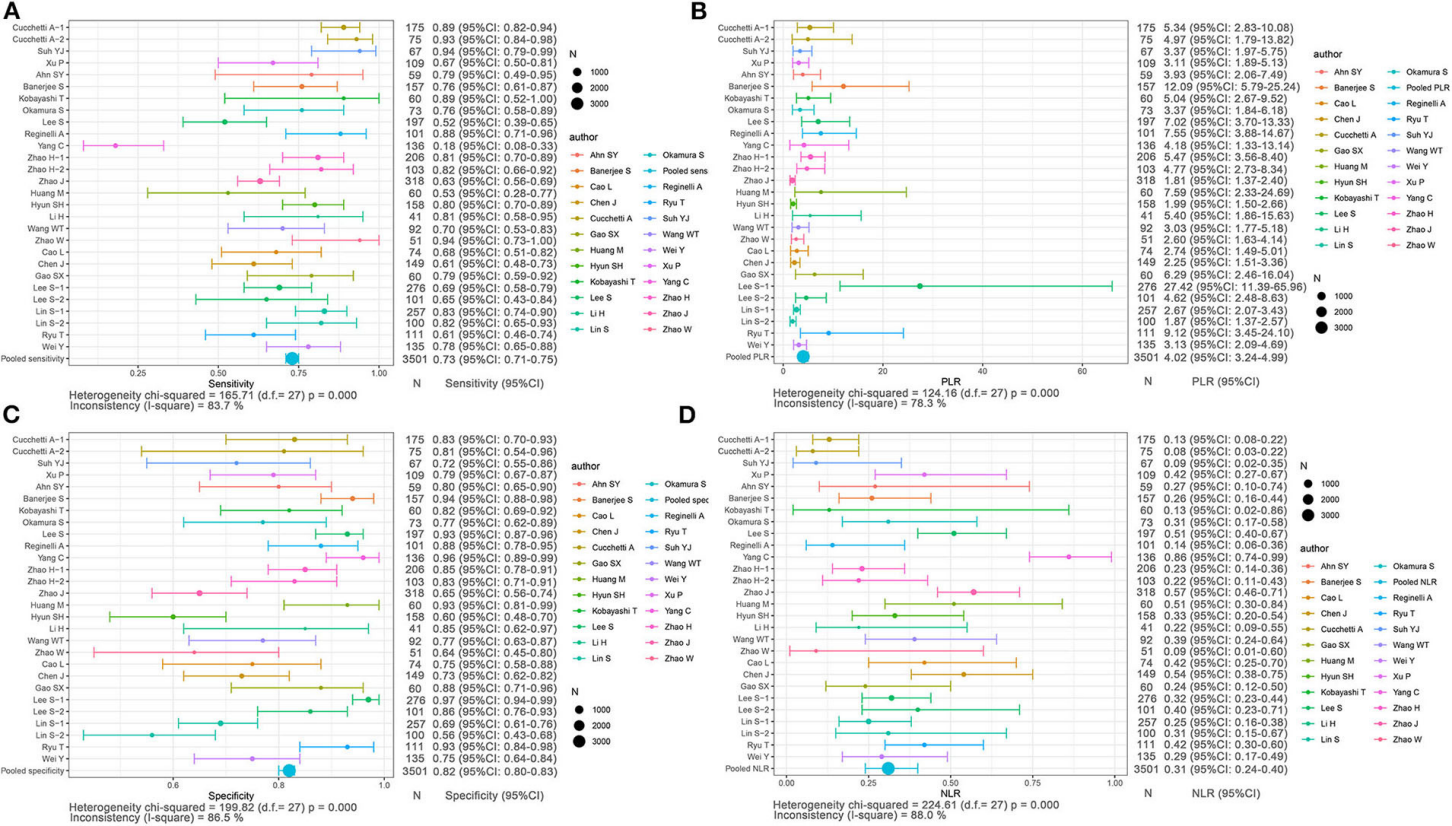
Forest plots based on non-radiomics for preoperative prediction of MVI in HCC. **(A)** Sensitivity, **(B)** PLR, **(C)** specificity, and **(D)** NLR.

Based on non-radiomics, 1,472 HCC patients were MVI-present and 2,029 were MVI-absent (Supplementary Table 2). The summarized sensitivity, specificity, PLR, and NLR of the non-radiomics group were 0.73 (95% CI: 0.71–0.75, *I*^2^ = 83.7%; Figure 4A), 0.82 (95% CI: 0.80–0.83, *I*^2^ = 86.5%; Figure 4C), 4.02 (95% CI: 3.24–4.99, *I*^2^ = 78.3%; Figure 4B), and 0.31 (95% CI: 0.24–0.40, *I*^2^ = 88.0%; Figure 4D), respectively. The sROC curve displayed the AUC of 0.8601 (**Figure 7B**).

### Subgroup Analysis of CT and MRI for Preoperative MVI Evaluation in HCC

The results of subgroup analysis based on CT and MRI are displayed in Table 4. CT showed the sensitivity, specificity, and AUC of 0.81 (95% CI: 0.75–0.86), 0.88 (95% CI: 0.84–0.91), and 0.9022, respectively, based on non-radiomics imaging, and the corresponding values were 0.79 (95% CI: 0.75–0.83), 0.79 (95% CI: 0.76–0.82), and 0.8640, respectively, on radiomics method. As for MRI, the non-radiomics imaging method displayed the sensitivity, specificity, and AUC of 0.67 (95% CI: 0.64–0.70), 0.81 (95% CI: 0.79–0.83), and 0.8269, respectively, and the corresponding values of the radiomics method were 0.86 (95% CI: 0.80–0.91), 0.79 (95% CI: 0.71–0.85), and 0.8829, respectively.

**Table 4:** Subgroup analysis of CT and MRI for preoperative MVI evaluation in HCC.

**Imaging machine**	**Category**	**Sensitivity**	**Specificity**	**PLR**	**NLR**	**AUC**
CT	Non-radiomics	0.81 (95% CI: 0.75–0.86)	0.88 (95% CI: 0.84–0.91)	6.37 (95% CI: 4.73–8.58)	0.23 (95% CI: 0.17–0.30)	0.9022
MRI	Non-radiomics	0.67 (95% CI: 0.64–0.70)	0.81 (95% CI: 0.79–0.83)	3.52 (95% CI: 2.75–4.50)	0.39 (95% CI: 0.30–0.50)	0.8269
CT	Radiomics	0.79 (95% CI: 0.75–0.83)	0.79 (95% CI: 0.76–0.82)	3.75 (95% CI: 3.15–4.47)	0.29 (95% CI: 0.20–0.41)	0.8640
MRI	Radiomics	0.86 (95% CI: 0.80–0.91)	0.79 (95% CI: 0.71–0.85)	3.92 (95% CI: 2.86–5.37)	0.19 (95% CI: 0.13–0.28)	0.8829

*CT, Computed tomography; MRI, Magnetic resonance imaging; PLR, Positive likelihood ratio; NLR, Negative likelihood ratio; AUC, Area under the curve*.

### Conventional Image Analysis for Preoperative MVI Evaluation in HCC

The included studies based on non-radiomics can be divided into a conventional imaging subgroup and a functional imaging subgroup. The former contained morphologic characteristics, such as tumor size, tumor capsule, margin, and enhancement. The latter included diffusion-weighted image (DWI), functional MRI, magnetic resonance spectrum, and digital image technology.

**Figure 5:**
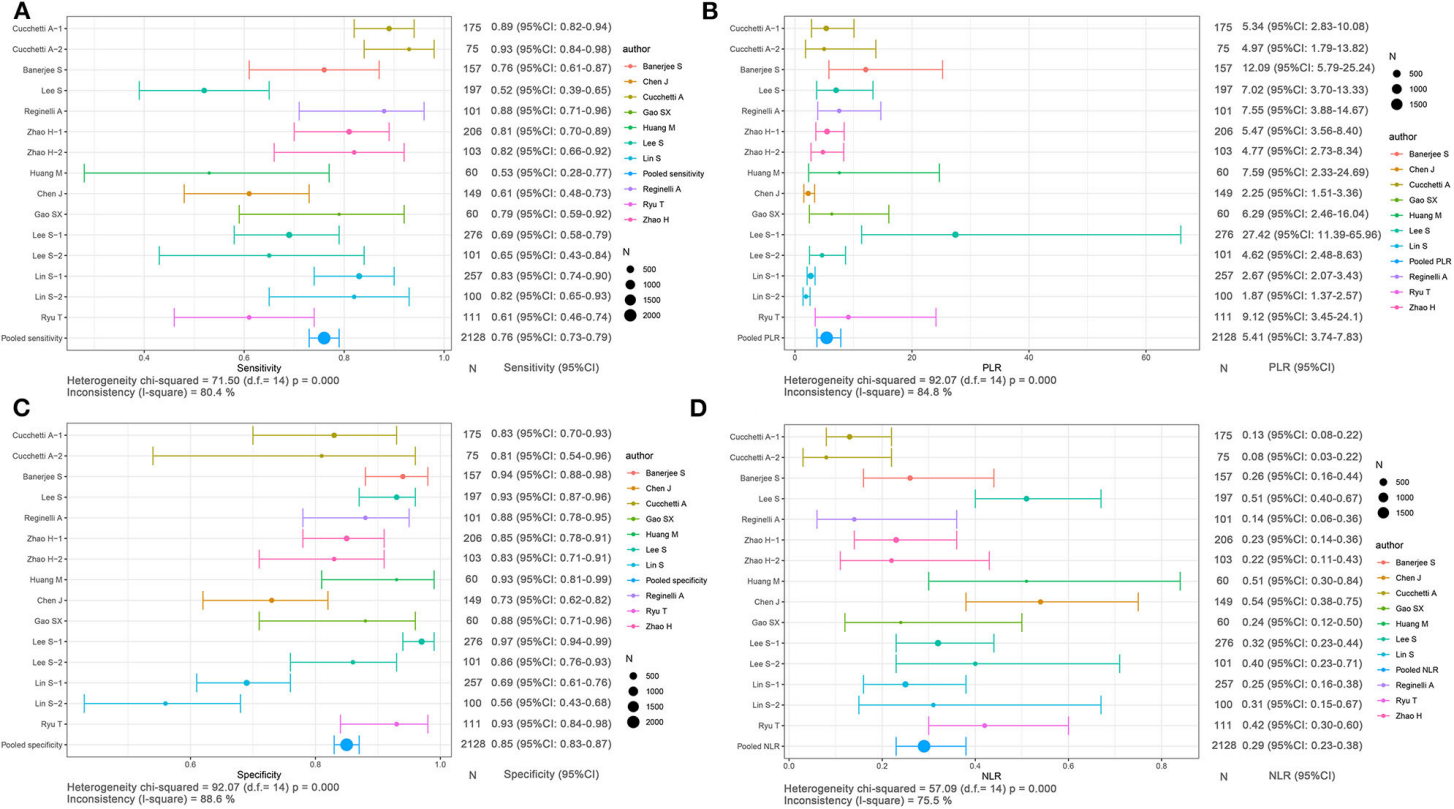
Forest plots based on conventional imaging analysis for preoperative prediction of MVI in HCC. **(A)** Sensitivity, **(B)** PLR, **(C)** specificity, and **(D)** NLR.

The integrated sensitivity, specificity, PLR, and NLR of the conventional imaging subgroup were 0.76 (95% CI: 0.73–0.79, *I*^2^ = 80.4%; Figure 5A), 0.85 (95% CI: 0.83–0.87, *I*^2^ = 88.6%; Figure 5C), 5.41 (95% CI: 3.74–7.83, *I*^2^ = 84.8%; Figure 5B), and 0.29 (95% CI: 0.23–0.38, *I*^2^ = 75.5%; Figure 5D), respectively. Conventional imaging for MVI prediction in HCC showed the AUC of 0.8794 on the sROC curve (**Figure 7C**).

### Functional Imaging Analysis for Preoperative MVI Evaluation in HCC

**Figure 6:**
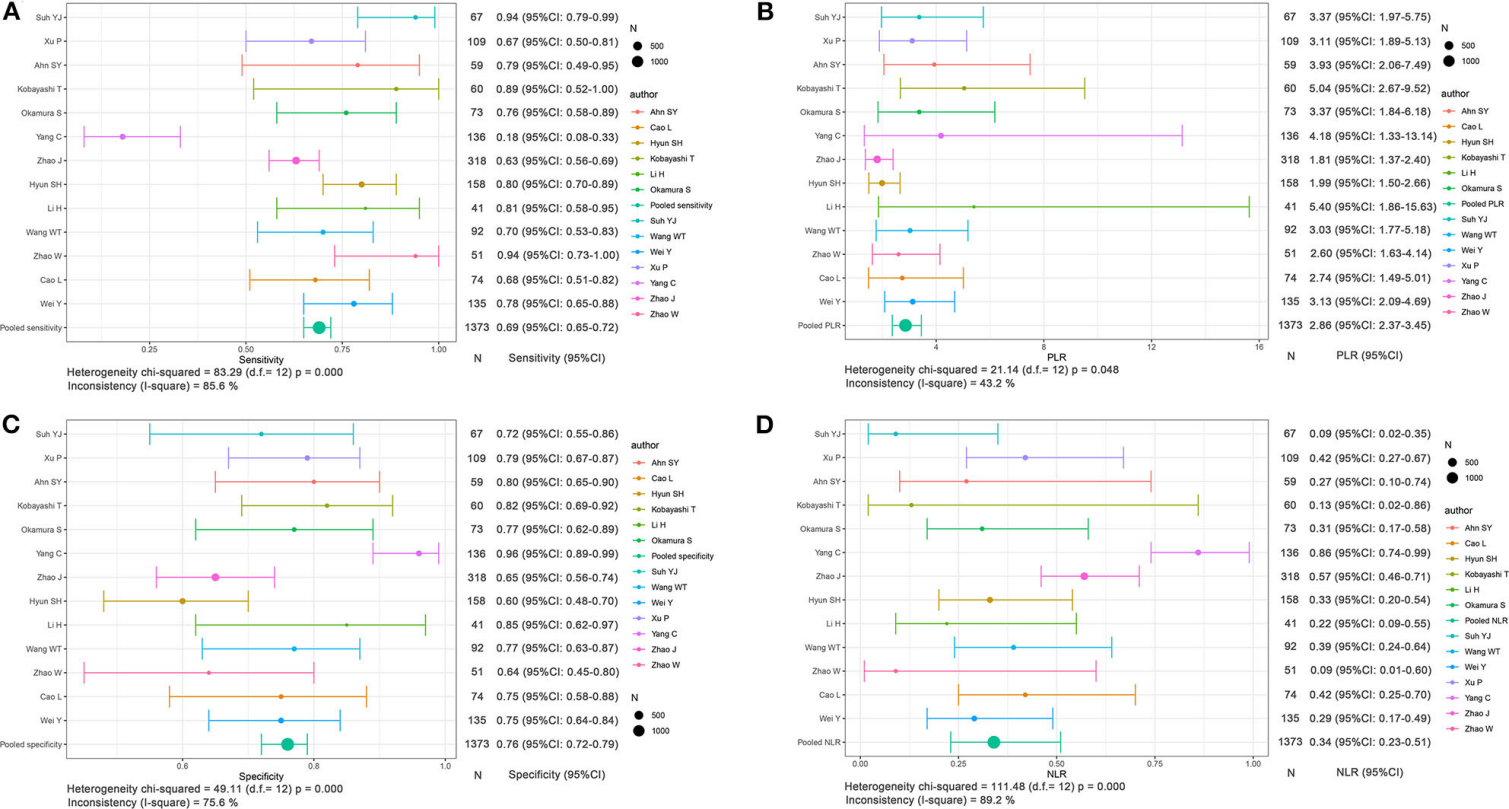
Forest plots based on functional imaging analysis for preoperative prediction of MVI in HCC. **(A)** Sensitivity, **(B)** PLR, **(C)** specificity, and **(D)** NLR.

**Figure 7:**
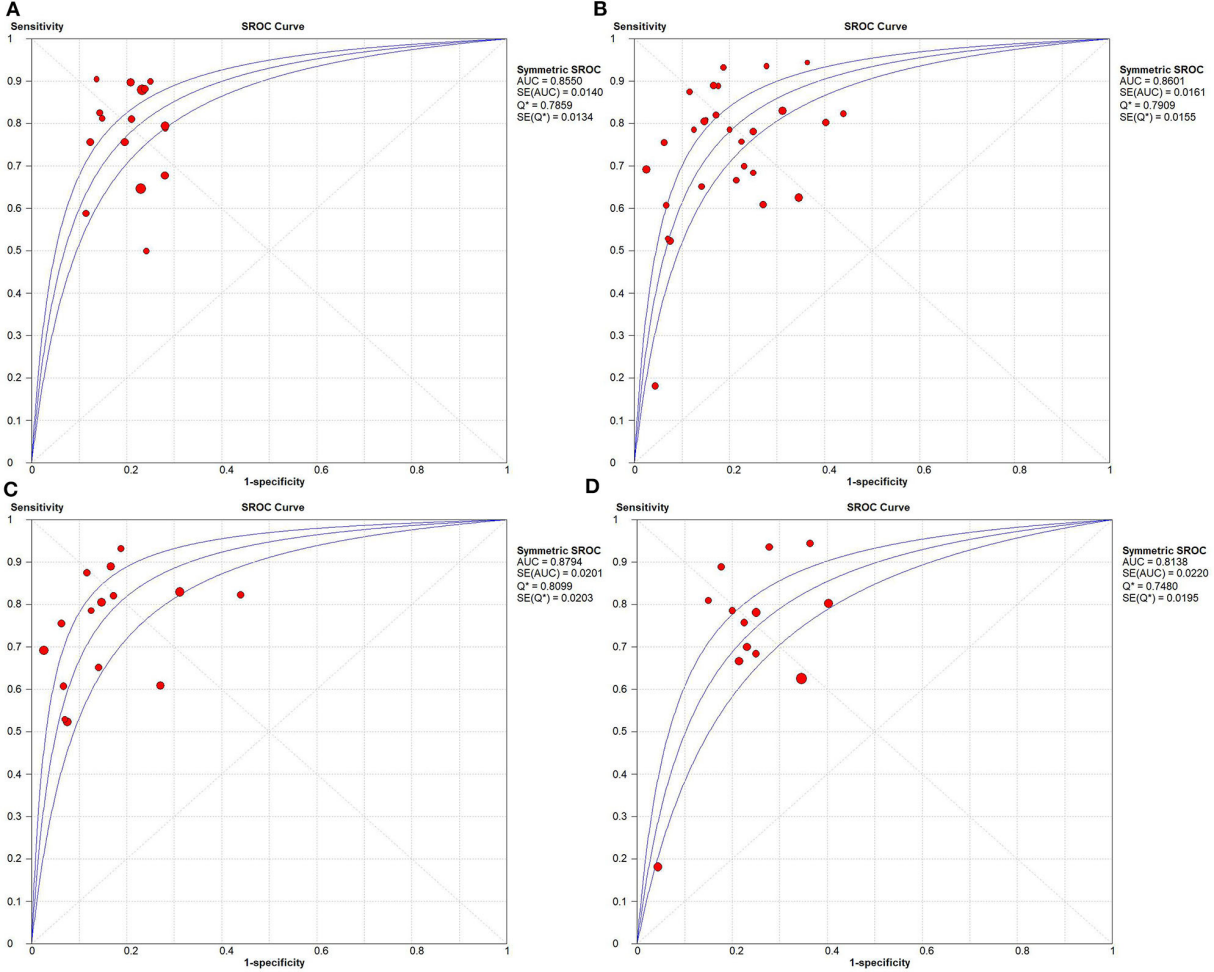
Summary receiver operating characteristic curves. **(A)** Radiomics, **(B)** non-radiomics, **(C)** conventional imaging analysis, and **(D)** functional imaging analysis.

The aggregated sensitivity, specificity, PLR, and NLR of the functional imaging subgroup were 0.69 (95% CI: 0.65–0.72, *I*^2^ = 85.6%; Figure 6A), 0.76 (95% CI: 0.72–0.79, *I*^2^ = 75.6%; Figure 6C), 2.86 (95% CI: 2.37–3.45, *I*^2^ = 43.2%; Figure 6B), and 0.34 (95% CI: 0.23–0.51, *I*^2^ = 89.2%; Figure 6D), respectively. The AUC of 0.8138 was less than that in the conventional imaging subgroup (Figure 7D).

## Discussion

We compared the diagnostic significance to preoperatively predict MVI status in HCC between radiomics and non-radiomics (conventional image, functional image) methods. The radiomics had a moderate diagnostic value to predict MVI status in HCC with pooled sensitivity, specificity, and AUC of 0.78, 0.78, and 0.8550, respectively. The aggregated sensitivity, specificity, and AUC based on non-radiomics were 0.73, 0.82, and 0.8601, respectively. These results showed that the radiomics method had a little higher sensitivity than the non-radiomics method for preoperative prediction of MVI status in HCC, although there was no difference in diagnostic value in terms of AUC. Another important finding was that radiomics had lower heterogeneity than non-radiomics for preoperative prediction of MVI status in HCC. The reasonable interpretation that may cause the heterogeneity in non-radiomics was partially that the image reviewing process depended partly on the subjective experience. In contrast, we considered that radiomics may be more potentially suitable for preoperative evaluation of MVI status in HCC patients.

Dataset (sample sizes of training cohort and validation cohort), modeling methods, imaging modality, feature number, and feature type all varied among these 33 included studies, which may influence the pooled sensitivity and specificity. Almost all studies in the radiomics group had training and validation cohorts to ensure the credibility of the results, and the varying sample sizes in different studies may more or less introduce sample size bias. As for modeling methods, most studies applied logistic regression because the MVI status (MVI present or absent) was a dichotomous variable, and mainstream analyzing methods like least absolute shrinkage and selection operator (LASSO) regression, support vector machine, and artificial neural network were also applied in these studies. Feature number and feature type in the included studies were derived from modeling methods. Lastly, different imaging modalities influenced the pooled sensitivity and specificity. In the radiomics group, MRI had higher predicted MVI than CT in terms of sensitivity, specificity, and AUC. In the non-radiomics group, CT had higher sensitivity, specificity, and AUC than MRI for MVI prediction. Despite the differences among imaging modalities, the numbers of studies in different subgroups were relatively small. Therefore, more studies focusing on each group of imaging modality are needed.

The current guideline of liver cancer diagnosis and treatment covers preoperative diagnosis, clinical staging, and post-therapeutic evaluation, which are implemented by radiologists and clinicians through visual observation of medical images and analysis of clinical information (7, 48, 49). Similarly, the Liver Imaging Reporting and Data System (LI-RADS) (50) is a standardization of the semantic imaging features of liver cancers, and involves a small portion of functional imaging, but lacks characteristic evaluation on tumor intrinsic heterogeneity that reflects different biological behaviors of HCC. Because the tumor imaging observed by the naked eye is limited by human visual perception, it cannot perceive certain subtle differences in the medical imaging data. Therefore, more medical imaging data should be fully explored and utilized. In recent years, artificial intelligence has been applied to analyze the enriched data contained in medical images, aiming to meet the increasing demands for individualized evaluation (51, 52). With the popularization of radiomics, different algorithms have been applied into modeling based on radiomics features for tumor phenotype prediction (53–56). A recent quantitative review of radiomics on HCC suggests that radiomics is a very promising non-invasive method for individualized evaluation based on intra-tumor heterogeneity analysis (57–59).

Reportedly, the radiomics models on MVI status prediction in HCC that are constructed on the basis of quantitative imaging features extracted from preoperative ultrasound, CT, or MRI images when applied with different algorithms demonstrate relatively high prediction performance (17–19, 24–29). Texture feature analysis was first used to demonstrate potential predictors of MVI status in HCC (17). Later, preoperative contrast-enhanced CT images of HCC at different phases were combined for extraction of image features, and the radiomics prediction model based on the least absolute shrinkage and selection operator algorithm well-performed MVI status in both the training group and the validation group (18, 24). A recent study suggests that LASSO plus GBDT is the optimal approach for MVI status evaluation in HCC among the diverse screening and modeling methods of image features (28). Moreover, the texture features extracted from preoperative contrast-enhanced MRI images at arterial and portal phases were also used to construct a predictive model of MVI status in HCC (29).

Recently, the radiomics features extracted from hepatobiliary-phase MRI images can improve the efficacy of MVI status prediction in HCC, given the clearer boundary of HCC in the hepatobiliary phase (26). In addition, two studies applying radiomics method to medical ultrasound images demonstrate that radiomics scores derived from contrast-enhanced ultrasound and multi-modal ultrasound images are independent predictors of MVI status in HCC (25, 27), and the latter performs better (25). Our study shows that the radiomics method, with relatively high prediction performance and consistency, can predict MVI non-invasively and provides more valuable information on clinical evaluation. In the future, prospective, multi-center, and large-scale studies are needed to confirm whether the radiomics features (or combined morphological features) extracted from preoperative images can work as MVI status predictors in HCC.

Multiple functional imaging parameters derived from DWI and PET/CT, including ADC, true diffusion coefficient, and mean apparent kurtosis coefficient, are considered valuable for MVI status evaluation in HCC. ADC originally based on the mono-exponential model of diffusion imaging is regarded as a valuable predictor (31, 32, 35, 39), probably because ADC reflects the decreased capillary perfusion in HCC accompanied with MVI (31, 32). Later, the true diffusion coefficient based on the intratraxel incoherent motion model was found superior over ADC for MVI status evaluation in HCC, which can truly reflect the molecular diffusion and microcirculation perfusion in the capillary network (15, 41, 42). Meanwhile, studies confirm that the mean apparent kurtosis coefficient based on the diffusion kurtosis imaging model is superior over the traditional ADC in evaluating the MVI status of HCC (14, 43), which reflects the more complicated microenvironment caused by MVI (14). Additionally, the 18F-FDG PET-CT reveals that the maximum uptake related to the uptake of tumor cells, and the standard intake ratio between tumor and normal liver (13), and the ratio between the maximum tumor uptake and the average uptake in normal liver (≥1.2) are all significantly associated with the MVI status in HCC (33). Taken together, our study indicates relatively high consistency in MVI status evaluation of HCC among these functional imaging studies, regardless of some problems in the way of our analysis, including interpretation of heterogeneity source. However, on the contrary from radiomics and conventional imaging studies, the smaller case number in the functional imaging subgroup may be limited in clinical applications due to lack of advanced MRI technology.

At present, the semantic features (e.g., peritumoral enhancement, non-smooth tumor margin, and peritumoral hypo-intensity at the hepatobiliary phase) derived from preoperative imaging may be potential independent predictors for MVI status in HCC (11, 12, 36, 40, 44, 45, 47). However, there is no consensus. Many studies focusing on MVI status prediction in HCC demonstrate that image features (e.g., tumor size, peritumoral enhancement, non-smooth tumor margin, peritumoral hypo-intensity at the hepatobiliary phase, intra-tumoral artery, and non-nodule type) are correlated with MVI status in HCC, which can be utilized to predict MVI status in HCC (10–12, 30, 36, 38, 40, 44–47). Among them, peritumor enhancement and non-smooth tumor margin are considered as important independent predictors in different studies (11, 44–46). However, mosaic architecture is the only independent predictor of MVI in Liver Imaging Reporting and Data System category 5 (LR-5) HCCs based on LI-RADS (44). The conflicting results can be partially attributed to the limited case numbers in different retrospective observational studies. Although these studies show certain correlation between macroscopic morphological features and microscopic pathological diagnosis, it is still difficult to establish a perfect interpretation between morphological features and pathophysiological changes of MVI status in HCC. In addition, the semantic features of these observations are non-uniform among different studies, and should be defined in a standardized way to realize the universality and reproducibility of image features. The factors mentioned above demonstrate that although the overall MVI status evaluation in HCC by semantic features is desirable, the relatively large deviation of evaluation performance may lead to unreliable results when applied with such prediction method. Finally, stable and effective imaging features with high predictive value should be discovered, standardized, and validated in further studies.

Our meta-analysis on the imaging methods in preoperative prediction of MVI status in HCC has two advantages. First, to the extent of our knowledge, this study involving 33 articles and 5,462 HCC cases is the first meta-analysis for preoperative assessment of MVI status in HCC patients by comparing radiomics, functional imaging, and conventional imaging methods. Second, radiomics based on machine learning algorithms is a new multidisciplinary branch in imageology and is widely used in medical image processing with rapid development. In this study, we have compared radiomics, functional imaging, and conventional imaging methods in evaluation of MVI status in HCC, which can facilitate the comparison of differences among imaging evaluation methods and provide references for subsequent studies and selection of clinical evaluations.

The drawbacks of this study around medical imaging MVI evaluation should be discussed. First, the pathological diagnosis of MVI status of HCC is the gold standard, but the specific pathological sampling and immunohistochemistry are not completely consistent in these studies, which will potentially lead to differences in judging MVI status of HCC and affect the consistency of the results. Second, image features have good predictive power for MVI status of HCC, but the subsequent treatment, recurrence, and survival analysis are not fully presented after enrollment. The value of imaging features used to evaluate the MVI status of HCC may be overestimated in prognostic stratification. Third, subjective image features (10) in a part of the studies, quantitative parameters in specific sequences (29), and concrete result interpretation steps (24) are inaccessible in certain studies, which makes a detailed subgroup quantitative judgement impossible.

## Conclusions

Our meta-analysis shows that preoperative imaging features are feasible to predict the MVI status of HCC and are potential biomarkers for postoperative recurrence of HCC. Radiomics method is more desirable than non-radiomics method, and possesses the objectivity of quantified features, high diagnostic efficacy, and high consistency among the studies.

## Data Availability Statement

All datasets generated for this study are included in the article/Supplementary Material.

## Author Contributions

SL, YD, and WL interpreted the study design. SL supervised our study. YD and WL obtained the research fund. JH screened the publications, performed statistics, and drafted the manuscript. WT and LZ screened the publications. QH helped perform statistics. SZ revised the first version of the manuscript. All authors contributed to the article and approved the submitted version.

## Conflict of Interest

The authors declare that the research was conducted in the absence of any commercial or financial relationships that could be construed as a potential conflict of interest.
