# Supplementary material for: Preoperative Prediction Power of Imaging Methods for Microvascular Invasion in Hepatocellular Carcinoma: A Systemic Review and Meta-Analysis
Source: Front Oncol. 2020 Jun 26;10:887. doi: 10.3389/fonc.2020.00887 (PMC7333535; doi:10.3389/fonc.2020.00887)
Supplement: Supplementary file 1 [file Table_1.DOCX]

Supplementary Table 1. Sensitivity analysis based on radiomics for preoperative prediction of MVI in HCC.

| Author | Sensitivity | Specificity | PLR | NLR | AUC |
| --- | --- | --- | --- | --- | --- |
| Zheng J | 0.77 (95%CI: 0.74-0.80) | 0.78 (95%CI: 0.76-0.81) | 3.55 (95%CI: 3.07-4.11) | 0.28 (95%CI: 0.21-0.36) | 0.8577 |
| Zheng J | 0.78 (95%CI: 0.75-0.81) | 0.78 (95%CI: 0.75-0.80) | 3.48 (95%CI: 3.02-4.01) | 0.27 (95%CI: 0.20-0.35) | 0.8498 |
| Peng J | 0.77 (95%CI: 0.74-0.80) | 0.79 (95%CI: 0.76-0.81) | 3.58 (95%CI: 3.09-4.15) | 0.28 (95%CI: 0.21-0.37) | 0.8602 |
| Peng J | 0.78 (95%CI: 0.75-0.81) | 0.78 (95%CI: 0.76-0.81) | 3.50 (95%CI: 3.02-4.06) | 0.28 (95%CI: 0.21-0.36) | 0.8552 |
| Xu X | 0.76 (95%CI: 0.73-0.79) | 0.79 (95%CI: 0.76-0.81) | 3.47 (95%CI: 2.96-4.07) | 0.30 (95%CI: 0.23-0.38) | 0.8543 |
| Xu X | 0.77 (95%CI: 0.74-0.80) | 0.78 (95%CI: 0.76-0.81) | 3.44 (95%CI: 2.97-3.97) | 0.29 (95%CI: 0.23-0.38) | 0.8522 |
| Ma X | 0.78 (95%CI: 0.75-0.80) | 0.78 (95%CI: 0.75-0.80) | 3.41 (95%CI: 3.00-3.87) | 0.28 (95%CI: 0.21-0.37) | 0.8453 |
| Ma X | 0.78 (95%CI: 0.75-0.81) | 0.78 (95%CI: 0.76-0.81) | 3.55 (95%CI: 3.10-4.07) | 0.26 (95%CI: 0.20-0.34) | 0.8593 |
| Zhu YJ | 0.77 (95%CI: 0.74-0.80) | 0.78 (95%CI: 0.76-0.81) | 3.49 (95%CI: 3.01-4.06) | 0.28 (95%CI: 0.21-0.37) | 0.8551 |
| Zhu YJ | 0.77 (95%CI: 0.74-0.80) | 0.78 (95%CI: 0.76-0.81) | 3.47 (95%CI: 3.02-4.00) | 0.28 (95%CI: 0.22-0.37) | 0.8528 |
| Ni M | 0.77 (95%CI: 0.74-0.80) | 0.78 (95%CI: 0.75-0.81) | 3.46 (95%CI: 3.01-3.97) | 0.28 (95%CI: 0.22-0.37) | 0.8515 |
| Feng ST | 0.77 (95%CI: 0.73-0.80) | 0.78 (95%CI: 0.76-0.81) | 3.51 (95%CI: 3.02-4.07) | 0.29 (95%CI: 0.23-0.38) | 0.8550 |
| Feng ST | 0.77 (95%CI: 0.74-0.80) | 0.78 (95%CI: 0.76-0.81) | 3.52 (95%CI: 3.04-4.07) | 0.29 (95%CI: 0.22-0.37) | 0.8551 |
| Hu HT | 0.80 (95%CI: 0.77-0.83) | 0.79 (95%CI: 0.76-0.81) | 3.64 (95%CI: 3.15-4.20) | 0.27 (95%CI: 0.20-0.35) | 0.8613 |
| Hu HT | 0.78 (95%CI: 0.75-0.81) | 0.79 (95%CI: 0.76-0.81) | 3.61 (95%CI: 3.18-4.09) | 0.27 (95%CI: 0.20-0.35) | 0.8608 |
| Yao Z | 0.77 (95%CI: 0.74-0.80) | 0.78 (95%CI: 0.76-0.81) | 3.47 (95%CI: 3.02-3.97) | 0.29 (95%CI: 0.22-0.37) | 0.8519 |

Abbreviations: positive likelihood ratio (PLR), negative likelihood ratio (NLR), area under the curve (AUC)
