# Supplementary material for: Preoperative Prediction Power of Imaging Methods for Microvascular Invasion in Hepatocellular Carcinoma: A Systemic Review and Meta-Analysis
Source: Front Oncol. 2020 Jun 26;10:887. doi: 10.3389/fonc.2020.00887 (PMC7333535; doi:10.3389/fonc.2020.00887)
Supplement: Supplementary file 2 [file Table_2.DOCX]

Supplementary Table 2. Sensitivity analysis based on non-radiomics for preoperative prediction of MVI in HCC.

| Author | Sensitivity | Specificity | PLR | NLR | AUC |
| --- | --- | --- | --- | --- | --- |
| Banerjee S | 0.73 (95%CI: 0.71-0.75) | 0.81 (95%CI: 0.79-0.83) | 3.85 (95%CI: 3.12-4.74) | 0.31 (95%CI: 0.24-0.40) | 0.8568 |
| Lee S | 0.74 (95%CI: 0.72-0.76) | 0.81 (95%CI: 0.79-0.83) | 3.93 (95%CI: 3.17-4.88) | 0.30 (95%CI: 0.23-0.39) | 0.8607 |
| Gao SX | 0.73 (95%CI: 0.71-0.75) | 0.82 (95%CI: 0.80-0.83) | 3.97 (95%CI: 3.20-4.94) | 0.31 (95%CI: 0.24-0.40) | 0.8589 |
| Cao L | 0.73 (95%CI: 0.71-0.75) | 0.82 (95%CI: 0.80-0.83) | 4.09 (95%CI: 3.28-5.11) | 0.30 (95%CI: 0.23-0.39) | 0.8631 |
| Chen J | 0.74 (95%CI: 0.71-0.76) | 0.82 (95%CI: 0.80-0.84) | 4.14 (95%CI: 3.31-5.18) | 0.30 (95%CI: 0.23-0.39) | 0.8645 |
| Lee S | 0.73 (95%CI: 0.71-0.76) | 0.80 (95%CI: 0.78-0.82) | 3.74 (95%CI: 3.08-4.55) | 0.31 (95%CI: 0.24-0.40) | 0.8557 |
| Lee S | 0.73 (95%CI: 0.71-0.75) | 0.81 (95%CI: 0.80-0.83) | 4.01 (95%CI: 3.21-5.00) | 0.30 (95%CI: 0.23-0.39) | 0.8612 |
| Wei Y | 0.73 (95%CI: 0.70-0.75) | 0.82 (95%CI: 0.80-0.84) | 4.09 (95%CI: 3.26-5.12) | 0.31 (95%CI: 0.24-0.40) | 0.8618 |
| Lin S | 0.72 (95%CI: 0.70-0.75) | 0.83 (95%CI: 0.81-0.84) | 4.14 (95%CI: 3.28-5.22) | 0.31 (95%CI: 0.24-0.40) | 0.8622 |
| Lin S | 0.73 (95%CI: 0.70-0.75) | 0.82 (95%CI: 0.81-0.84) | 4.17 (95%CI: 3.35-5.17) | 0.31 (95%CI: 0.24-0.40) | 0.8647 |
| Ryu T | 0.73 (95%CI: 0.71-0.76) | 0.81 (95%CI: 0.79-0.83) | 3.93 (95%CI: 3.17-4.87) | 0.30 (95%CI: 0.23-0.39) | 0.8597 |
| Li H | 0.73 (95%CI: 0.71-0.75) | 0.82 (95%CI: 0.80-0.83) | 4.00 (95%CI: 3.21-4.97) | 0.31 (95%CI: 0.24-0.40) | 0.8591 |
| Zhao W | 0.73 (95%CI: 0.70-0.75) | 0.82 (95%CI: 0.80-0.84) | 4.11 (95%CI: 3.29-5.14) | 0.31 (95%CI: 0.24-0.40) | 0.8580 |
| Huang M | 0.73 (95%CI: 0.71-0.76) | 0.81 (95%CI: 0.80-0.83) | 3.97 (95%CI: 3.20-4.93) | 0.30 (95%CI: 0.23-0.39) | 0.8604 |
| Hyun SH | 0.73 (95%CI: 0.70-0.75) | 0.83 (95%CI: 0.81-0.84) | 4.16 (95%CI: 3.34-5.19) | 0.31 (95%CI: 0.24-0.40) | 0.8651 |
| Wang WT | 0.73 (95%CI: 0.71-0.75) | 0.82 (95%CI: 0.80-0.83) | 4.08 (95%CI: 3.26-5.10) | 0.30 (95%CI: 0.23-0.40) | 0.8626 |
| Zhao J | 0.75 (95%CI: 0.72-0.77) | 0.83 (95%CI: 0.81-0.84) | 4.18 (95%CI: 3.36-5.20) | 0.30 (95%CI: 0.23-0.39) | 0.8662 |
| Reginelli A | 0.73 (95%CI: 0.70-0.75) | 0.81 (95%CI: 0.80-0.83) | 3.92 (95%CI: 3.16-4.86) | 0.32 (95%CI: 0.25-0.41) | 0.8556 |
| Zhao H | 0.73 (95%CI: 0.70-0.75) | 0.81 (95%CI: 0.80-0.83) | 3.96 (95%CI: 3.19-4.93) | 0.31 (95%CI: 0.24-0.40) | 0.8583 |
| Zhao H | 0.73 (95%CI: 0.70-0.75) | 0.82 (95%CI: 0.80-0.83) | 4.00 (95%CI: 3.21-4.99) | 0.31 (95%CI: 0.24-0.40) | 0.8588 |
| Yang C | 0.75 (95%CI: 0.72-0.77) | 0.81 (95%CI: 0.79-0.83) | 4.02 (95%CI: 3.23-5.00) | 0.31 (95%CI: 0.26-0.37) | 0.8597 |
| Okamura S | 0.73 (95%CI: 0.71-0.75) | 0.82 (95%CI: 0.80-0.83) | 4.06 (95%CI: 3.25-5.07) | 0.31 (95%CI: 0.24-0.40) | 0.8615 |
| Kobayashi T | 0.73 (95%CI: 0.71-0.75) | 0.82 (95%CI: 0.80-0.83) | 4.00 (95%CI: 3.20-4.98) | 0.31 (95%CI: 0.24-0.40) | 0.8583 |
| Ahn SY | 0.73 (95%CI: 0.71-0.75) | 0.82 (95%CI: 0.80-0.83) | 4.03 (95%CI: 3.23-5.03) | 0.31 (95%CI: 0.24-0.40) | 0.8604 |
| Xu P | 0.73 (95%CI: 0.71-0.75) | 0.82 (95%CI: 0.80-0.83) | 4.08 (95%CI: 3.26-5.10) | 0.30 (95%CI: 0.23-0.39) | 0.8628 |
| Suh YJ | 0.73 (95%CI: 0.70-0.75) | 0.82 (95%CI: 0.80-0.83) | 4.06 (95%CI: 3.25-5.08) | 0.32 (95%CI: 0.25-0.41) | 0.8564 |
| Cucchetti A | 0.71 (95%CI: 0.69-0.74) | 0.82 (95%CI: 0.80-0.83) | 3.98 (95%CI: 3.20-4.95) | 0.32 (95%CI: 0.25-0.41) | 0.8550 |
| Cucchetti A | 0.72 (95%CI: 0.70-0.74) | 0.82 (95%CI: 0.80-0.83) | 4.00 (95%CI: 3.22-4.98) | 0.32 (95%CI: 0.25-0.41) | 0.8551 |

Abbreviations: positive likelihood ratio (PLR), negative likelihood ratio (NLR), area under the curve (AUC)
